# IgG4-related lung disease presenting as interstitial lung disease with bronchiolitis: A case report

**DOI:** 10.1097/MD.0000000000009140

**Published:** 2017-12-08

**Authors:** Chiu-Fan Chen, Kuo-An Chu, Yen-Chiang Tseng, Chang-Che Wu, Ruay-Sheng Lai

**Affiliations:** a Division of Chest Medicine, Department of Internal Medicine; b Division of Thoracic Surgery, Department of Surgery; c Institute of Clinical Medicine, National Yang-Ming University, Taipei; d Department of Pathology and Laboratory Medicine, Kaohsiung Veterans General Hospital, Kaohsiung, Taiwan; e Faculty of Medicine, National Yang-Ming University, Taipei, Taiwan.

**Keywords:** bronchiolitis, IgG4, interstitial lung disease

## Abstract

**Rationale:**

IgG4-related disease is a rare and novel disease entity that tends to involve multiple organs. The pulmonary manifestation of this disease is highly variable and may mimic lung cancer, pneumonia, interstitial lung disease (ILD), sarcoidosis, and so forth. Small airway disease is rarely reported in IgG4-related lung disease (IgG4-RLD). In the current study, we describe a rare case of IgG4-RLD with patterns of ILD and bronchiolitis.

**Patient concern:**

A 43-year-old man had chronic cough and dyspnea on exertion for 4 years. Initial chest radiography showed diffuse interstitial infiltration. Follow-up chest computed tomography 4 years later revealed bilateral diffuse centrilobular nodules with tree-in-bud pattern, bronchial wall thickening, and mediastinal lymph nodes. Bilateral diffuse multifocal ground-glass opacities and mosaic attenuation were also observed. Pulmonary function test revealed mixed restrictive and obstructive ventilatory impairment.

**Diagnoses:**

Video-assisted thoracoscopic surgery (VATS) lung biopsy showed interstitial fibrosis with lymphoplasmacytic infiltration rich in IgG4-positive plasma cells. Serum IgG4 level also showed remarkable elevation. Therefore, IgG4-RLD is confirmed.

**Intervention:**

VATS wedge resection of right upper lobe and mediastinal lymph node.

**Outcomes:**

The patient responded well to steroid and immunosuppression therapy, and was regular followed-up in outpatient clinic.

**Lessons:**

IgG4-RLD should be considered not only in ILD, but also in small airway disease. Serum IgG4 level may be a useful tool for screening.

## 1 Introduction

IgG4-related disease (IgG4-RD) is a novel recognized fibro-inflammatory disorder and usually manifests as a mass-like lesion or generalized swelling in various organs, with characteristic pathological findings showing IgG4-rich plasma cell infiltration, chronic inflammation, and fibrosis. Nearly any organ can be involved, and many cases have elevated serum IgG4 concentrations.^[1]^ Autoimmune pancreatitis, sialadenitis, and dacryoadenitis are common and typical presentations of this unique disease.^[2]^ Although the lung is occasionally involved in IgG4-RD, the clinical and image presentations are highly variable, with a disease spectrum involving mediastinal lymphadenopathy, interstitial pneumonia, pleural effusion, and uncommonly, airway disease.^[3]^ Small airway disease is a rare presentation in this disease entity. In this report, we present a biopsy-proven case of IgG4-related lung disease (IgG4-RLD) with interstitial lung disease and bronchiolitis.

## 2 Case report

Written informed consent was obtained from the patient for publication of the article.

**Figure 1:**
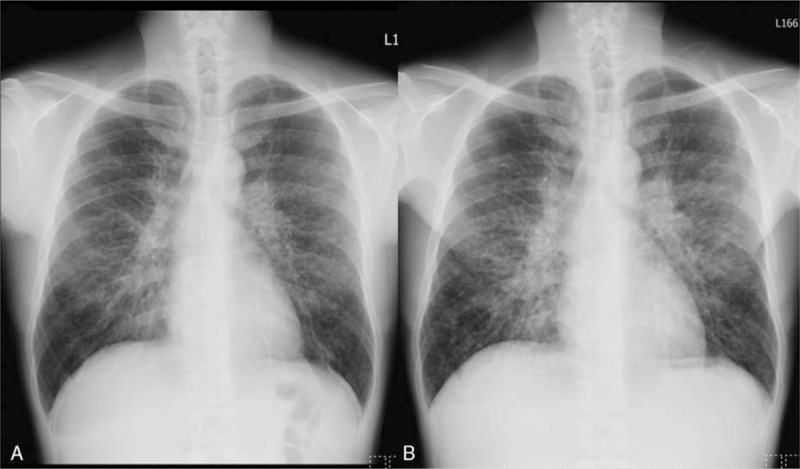
(A) Initial chest radiograph showing bilateral hilar enlargement and diffuse reticular-nodular pattern with central predominance. (B) Chest radiograph 4 years later showing progression of bilateral diffuse reticulo-nodular pattern.

**Figure 2:**
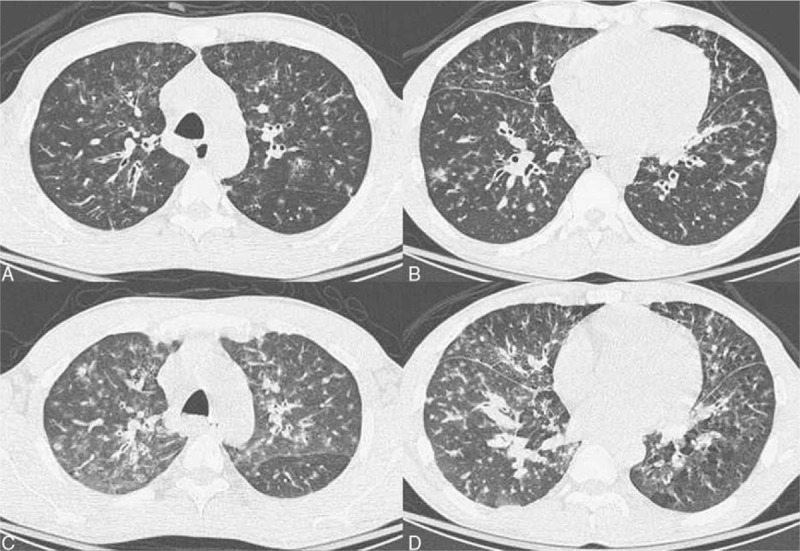
Chest computed tomography during inspiration showing bilateral diffuse centrilobular nodules, tree-in-bud pattern, and bronchial wall thickening (A, B: inspiration). Bilateral multifocal ground-glass opacities and mosaic pattern, indicating air trapping, were observed during expiration (C, D: expiration).

A 43-year-old man presented with chronic exertional dyspnea and cough for 4 years. The initial chest radiograph (Fig. 1A) showed bilateral diffuse reticulo-nodular opacities with central predominance 4 years before, suggesting interstitial lung disease (ILD). He was a teacher and denied systemic diseases or smoking. He refused surgical lung biopsy at first and then was lost to follow-up. Progressively worsening dyspnea bothered him in the past 4 years. Until the recent 3 months, the dyspnea and cough significantly worsened, so he revisited our hospital. He had no fever or purulent sputum. Chest radiography revealed progression of bilateral diffuse reticulo-nodular opacities (Fig. 1B). The room air saturation was 94% at rest, and the other physical examinations were not remarkable. Laboratory examination revealed the following values: white blood cells, 10,500/μL; hemoglobin, 7.5 g/dL; platelet count, 583,000/μL; C-reactive protein, 7.31 mg/dL; eosinophil, 12%; and total IgE, 1823 IU/mL. The rheumatoid factor showed a borderline result (21.3 IU/mL; normal range, <20 IU/mL), and other autoimmune markers were negative. The results of the pulmonary function test were as follows: forced vital capacity (FVC), 3.24 L (79% predicted); forced expiratory volume in 1 second (FEV1), 2.19 L (64.4% predicted); FEV1/FVC, 67.6%; total lung capacity (TLC), 5.02 L (79.2% predicted); residual volume (RV), 1.89 L (98.4% predicted); and RV/TLC, 37.6% (124.3% predicted). The diffusing capacity of the lung for carbon monoxide was 23.7 mL/(min mmHg) (80.9% predicted). Mixed restrictive and obstructive ventilatory impairment was suggested. Microbial cultures of sputum were negative. Chest computed tomography (CT) revealed bilateral diffuse centrilobular nodules, tree-in-bud pattern, bronchial wall thickening, and multifocal ground-glass opacities. Some mediastinal lymph node enlargement and focal consolidation over the bilateral lower lung were also observed. Multiple mosaic attenuation that suggested air trapping was found especially in the expiratory phase (Fig. 2).

**Figure 3:**
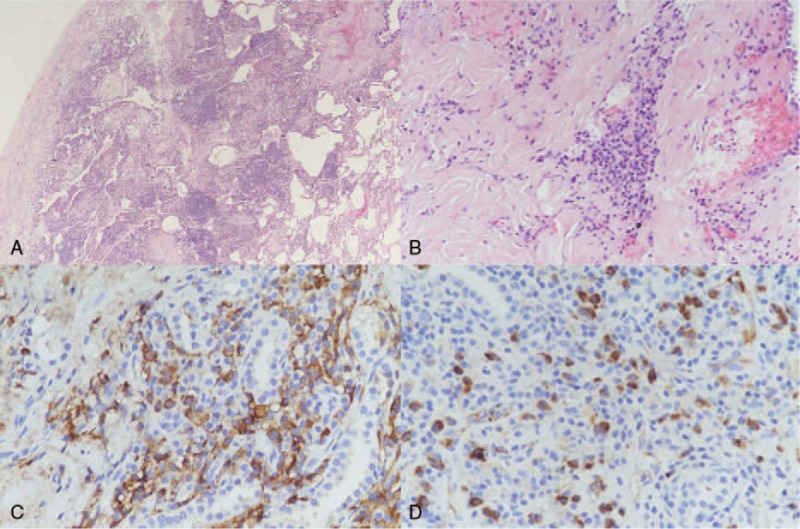
The pathological features of the surgically resected lung tissue shows interstitial infiltration with plasma cells, lymphocytes, and mild eosinophils infiltration and interstitial fibrosis (A: H&E staining, original magnification ×40; B: H&E staining, original magnification ×200). The IHC shows IgG4-positive plasma cells of >50/HPF and a IgG4/IgG-positive plasma cell ratio of >40% (C: IHC for IgG original magnification ×400; D: IHC for IgG4 original magnification ×400). H&E = hematoxylin and eosin, HPF = high-power field, IHC = immunohistochemistry.

Initial differential diagnosis includes diffuse panbronchiolitis, sarcoidosis and hypersensitivity pneumonitis. Connective tissue related ILD was excluded because of no associated symptoms such as arthralgia or skin rash, and there was no remarkable elevation of autoimmune marker. Pulmonary infection including tuberculosis was also unlikely due to no infection sign, negative sputum culture, and acid-fast stain. The patient received video-assisted thoracoscopic wedge resection of the right upper lobe and lymph node dissection. Both pathological findings showed prominent interstitial lymphoplasmacytic infiltrates, mild eosinophil infiltration, and fibrosis involving the alveolar septa. On immunohistochemistry, the number of IgG4-positive plasma cells was >50/high-power field (HPF), and the IgG4/IgG-positive plasma cell ratio was >40% (Fig. 3). The serum concentrations of IgG (5240 mg/dL; normal range, 751–1560 mg/dL) and IgG4 (1490 mg/dL; normal range, 3.92–86.4 mg/dL) also showed remarkable elevation. IgG4-RLD with bronchiolitis was confirmed. The abdominal CT and Gallium-67 inflammation scan did not revealed pancreatitis or abnormal finding in other organ. There were also no symptoms regarding salivary gland or lacrimal gland.

We started anti-inflammatory treatment with prednisolone 20 mg/day and azathioprine 50 mg/day, and his respiratory symptoms and chest image improved after 3 months of treatment. The eosinophilia and anemia also improved. His serum IgG4 level gradually decreased but was still higher than the upper normal limit. The follow-up pulmonary function test revealed the following results: FEV1, 2.55 L (73.1% predicted); FEV1/FVC, 75%; TLC, 7.05 L (111.2% predicted); RV, 3.42 L (178.1% predicted); RV/TLC, 48.5% (160.2% predicted). Currently, he is in stable condition and regularly followed up in the outpatient clinic.

## 3 Discussion

The IgG4 antibody is a subclass of IgG and accounts for <5% of the total IgG in normal conditions. IgG4 was traditionally thought to be an anti-inflammatory immunoglobulin because of its inability to activate classical complement pathways, aggregate antigens, and form immune complexes.^[1]^ IgG4 elevation in association with autoimmune pancreatitis was first recognized in 2001^[4]^ and, later on, was also described in various idiopathic diseases which was previously thought to be confined to a single organ.^[1,2]^ In the early 2010 s, the understanding of IgG4-RD had become more comprehensive. Now, it is considered as a systemic disease and usually presents with multiple-organ involvement and, typically, with mass formation or diffuse enlargement of the affected organs. Autoimmune pancreatitis, sialadenitis, dacryoadenitis, and retroperitoneal fibrosis are the most common and typical presentation of IgG4-RD. Multiple-organ involvement is common, and nearly all organs can be involved by this unique disease, although the prevalence of each organ involvement is still unclear.^[1,2]^ The pathogenesis of IgG4-RD remained unclear. Whether the excess of IgG4 antibodies results in tissue destruction and fibrosis, or is merely a disease marker in response to the inflammation needs to be elucidated. Many patients are associated with allergic diseases such as asthma and atopy. Eosinophilia and elevated serum IgE level were also observed in 40% of patients. Therefore, IgG4-RD is suggested to be possibly associated with autoimmunity.^[1]^

The definite diagnosis of IgG4-RD must rely on histopathological analysis. The key morphological features are dense lymphoplasmacytic infiltrates, storiform fibrosis, obliterative phlebitis, and mild-to-moderate eosinophil infiltrates. However, the typical storiform fibrosis may not be apparent in the lung.^[3]^ Increase of IgG4 positive plasma cells in tissue is helpful for diagnosis, especially when correlating with the morphological features on pathological examination.^[1]^ Elevated serum IgG4 concentration is also a useful screening tool but is not reliable enough to make a diagnosis. In a meta-analysis conducted by Hao et al,^[5]^ the pooling accuracy of serum IgG4 levels of >135–144 mg/dL yielded a sensitivity of 87% and specificity of 83% for diagnosing IgG4-RD. Serum IgG4 level may also increase in other diseases such as bronchiectasis, asthma, idiopathic pulmonary fibrosis, emphysema, hypersensitivity pneumonitis, primary sclerosing cholangitis, inflammatory bowel disease, Hashimoto's thyroiditis, and in approximately 5% of the normal population.^[1,6]^ As IgG4-RD may have some histological variations between different organs, Japanese experts proposed comprehensive diagnostic criteria for this disease, which are practical for general physicians and nonspecialists. Patients who fulfill the following 3 criteria are diagnosed as having IgG4-RD: organ involvement or damage consistent with IgG4-RD; serum IgG4 level of >135 mg/dL; and histopathology showing marked lymphoplasmacytic infiltration, fibrosis, IgG4/IgG-positive cells of >40%, and >10 IgG4-positive cells per high power field.^[7]^ Our case fulfilled all 3 comprehensive diagnostic criteria for IgG4-RD and involved only the lung; therefore, the diagnosis of IgG4-RLD was made.

The percentage of pulmonary involvement in IgG4-RD is uncertain. According to previous literatures, lung involvement was occasionally found in IgG4-RD patients. Pulmonary involvement of IgG4-RD is highly variable and can mimic lung cancer, usual interstitial pneumonia, nonspecific interstitial pneumonia, organizing pneumonia, and sarcoidosis. Four patterns were observed in IgG4-RLD, which include mediastinal, parenchymal, pleural, and airway involvement. Mediastinal and hilar lymphadenopathy are the most common pulmonary patterns. Parenchymal involvement includes nodules, masses, ground-glass opacities, consolidation, reticular opacities, bronchovascular thickening, and even honeycombing. Pleural effusion or nodules as predominant presentations were also reported but were uncommon. Airway involvement in IgG4-RLD is rare, including tracheobronchial stenosis and traction bronchiectasis.^[3]^

The bronchiolitis pattern of IgG4-RLD is unique. To our knowledge, only 6 cases of IgG4-RD with bronchiolitis were reported in literature, and all of them were Japanese patients (Table 1).^[8–12]^ Our case is the first IgG4-RLD with bronchiolitis in Taiwan, and the mosaic attenuation hasn’t been reported previously. The bronchial thickening and centrilobular nodules on chest CT are also clear evidences of large and small airway narrowing. The pulmonary function test of our patient showed mixed obstructive and restrictive pattern, which was compatible to ILD and small airway disease. However, similar to 2 previously reported cases,^[11–12]^ our case also had normal DLCO value, and we suggest this is because of more extent of small airway involvement than interstitium.

**Table 1:**
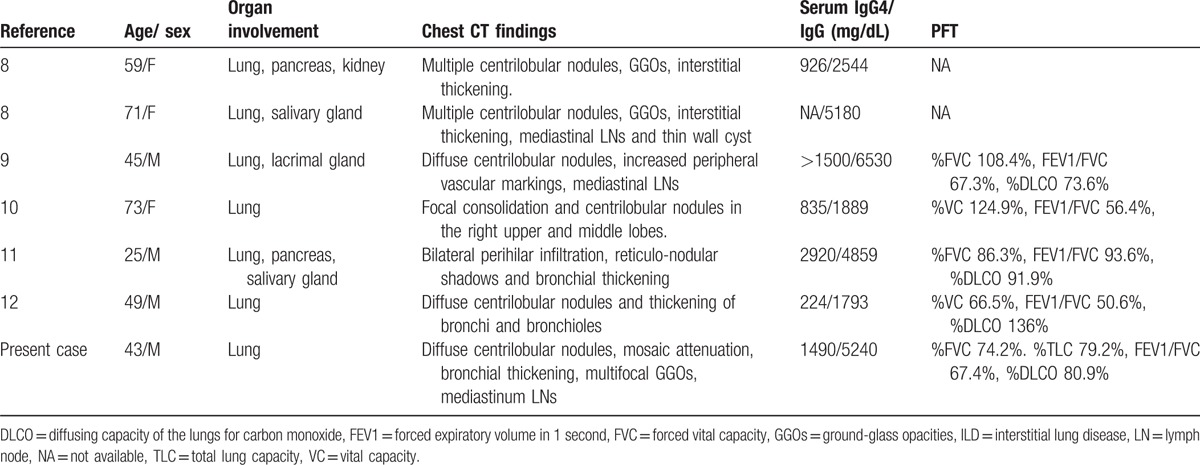
Summary of published cases of IgG4-related lung disease with bronchiolitis.

Steroid therapy effectively relieved our patient's dyspnea and pulmonary lesions. The initial mixed restrictive and obstructive pattern in the pulmonary function test also resolved after steroid therapy. The mainstay treatment for IgG4-RLD is similar to that for IgG4-RD, which is corticosteroid therapy, and most patients have favorable response in 2 weeks. The usual dose for oral prednisone is between 0.6 and 1 mg/(kg·day) for 2 to 4 weeks, and then the dose is gradually tapered every 2 to 4 weeks to maintain a dose of 2.5 to 5 mg/day and then discontinued within 3 years.^[3,6]^ However, some patients may not achieve complete resolution and relapse may occur. For steroid-refractory patients, immunosuppressants such as azathioprine, mycophenolate mofetil, and cyclosporine can be considered. Anti CD20 antibody (Rituximab) is also a reasonable treatment choice for refractory cases. The long-term survival in IgG4-RLD remains unclear because of the limited clinical follow-up data.^[3,6]^

This is the first case of IgG4-RLD with bronchiolitis in Taiwan. In conclusion, chest physicians should keep IgG4-RLD in mind because this novel disease is frequently underdiagnosed but potentially treatable.^[2]^ This disease should be considered not only in ILD, but also in small airway disease. Elevation of serum IgG4 level and associated multiorgan involvement are useful clinical clues, but definitive diagnosis requires surgical biopsy.
